# Diaqua­bis­(4-carb­oxy-2-ethyl-1*H*-imidazole-5-carboxyl­ato-κ^2^ *N* ^3^,*O* ^4^)zinc *N*,*N*-dimethyl­formamide disolvate

**DOI:** 10.1107/S1600536811012992

**Published:** 2011-04-13

**Authors:** Cheng-Jun Hao, Hui Xie

**Affiliations:** a College of Chemistry and Chemical Engineering, Pingdingshan University, Pingdingshan 467000, People’s Republic of China.

## Abstract

In the title compound, [Zn(C_7_H_7_N_2_O_4_)_2_(H_2_O)_2_]·2C_3_H_7_NO, the Zn^II^ ion, which lies on a center of inversion, is coordinated by two O atoms and two N atoms from two 4-carboxy-2-ethyl-1*H*-imid­azole-5-carboxyl­ato anions and two water O atoms in an octa­hedral environment, Each 4-carboxy-2-ethyl-1*H*-imid­azole-5-carboxyl­ato ligand adopts a bidentate chelating mode to the Zn^II^ ion, forming two five-membered metalla rings. In the crystal, a two-dimensional framework parallel to (010) is formed by N—H⋯O and O—H⋯O hydrogen bonds.

## Related literature

For the properties of complexes derived from imidazole-4,5-dicarboxylic acid, see: Maji *et al.* (2005 ▶); Yang & Zhang (2006 ▶). For our previous work based on 2-ethyl-4,5-imidazole­dicarboxyl­ate, see: Tian *et al.* (2010 ▶).
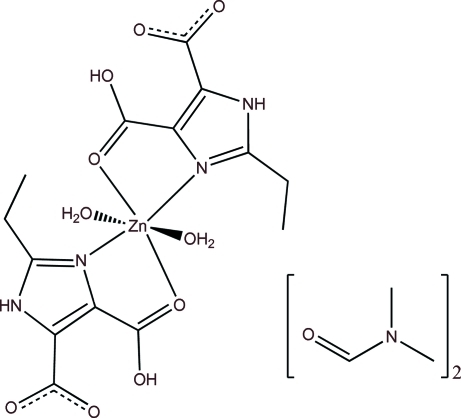


## Experimental

### 

#### Crystal data

[Zn(C_7_H_7_N_2_O_4_)_2_(H_2_O)_2_]·2C_3_H_7_NO
*M* *_r_* = 613.89
Monoclinic, 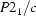

*a* = 7.2817 (8) Å
*b* = 20.660 (2) Å
*c* = 9.3623 (9) Å
β = 111.846 (7)°
*V* = 1307.3 (2) Å^3^
*Z* = 2
Mo *K*α radiation
μ = 1.01 mm^−1^
*T* = 296 K
0.53 × 0.41 × 0.31 mm

#### Data collection

Bruker APEXII area-detector diffractometer
Absorption correction: multi-scan (*SADABS*; Sheldrick, 1996 ▶) *T*_min_ = 0.616, *T*_max_ = 0.744
10742 measured reflections
2619 independent reflections
2083 reflections with *I* > 2σ(*I*)
*R*_int_ = 0.037

#### Refinement

*R*[*F* ^2^ > 2σ(*F* ^2^)] = 0.034
*wR*(*F* ^2^) = 0.086
*S* = 1.02
2619 reflections
181 parameters
3 restraints
H-atom parameters constrained
Δρ_max_ = 0.22 e Å^−3^
Δρ_min_ = −0.36 e Å^−3^

### 

Data collection: *APEX2* (Bruker, 2007 ▶); cell refinement: *SAINT* (Bruker, 2007 ▶); data reduction: *SAINT*; program(s) used to solve structure: *SHELXTL* (Sheldrick, 2008 ▶); program(s) used to refine structure: *SHELXTL*; molecular graphics: *SHELXTL*; software used to prepare material for publication: *SHELXTL*.

## Supplementary Material

Crystal structure: contains datablocks I, global. DOI: 10.1107/S1600536811012992/nk2096sup1.cif

Structure factors: contains datablocks I. DOI: 10.1107/S1600536811012992/nk2096Isup2.hkl

Additional supplementary materials:  crystallographic information; 3D view; checkCIF report

## Figures and Tables

**Table 1:** Hydrogen-bond geometry (Å, °)

*D*—H⋯*A*	*D*—H	H⋯*A*	*D*⋯*A*	*D*—H⋯*A*
O1*W*—H1*W*⋯O1^i^	0.85	1.95	2.798 (2)	173
O1*W*—H2*W*⋯O1^ii^	0.85	2.06	2.894 (2)	168
O3—H3⋯O2	0.85	1.62	2.473 (2)	177
N2—H2⋯O5	0.86	1.85	2.689 (2)	166

Symmetry codes: (i) 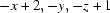; (ii) 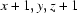.

**Fig. 1.**
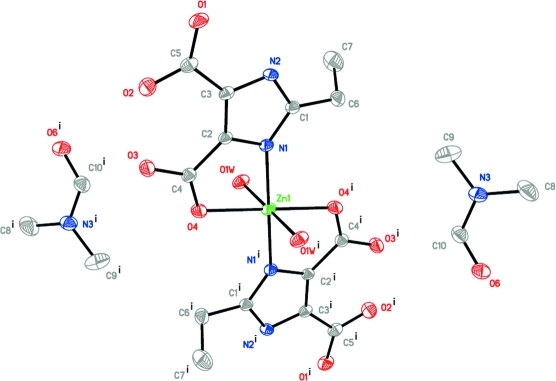
The structure of the title compound, with 30% probability displacement ellipsoids (H atoms are omitted for clarity). [Symmetry codes: (i) -x + 2, -y, -z + 1.]

## supplementary crystallographic information

### Comment

Imidazole-4,5-dicarboxylic acid (H_3_Imda) can be deprotonated to generate three types of anions, namely Imda^3-^, HImda^2-^ and H_2_Imda^-^and react with metal ions to form fascinating structures with different structures and useful properties (Maji *et al.*, 2005; Yang *et al.*, 2006). In previous studies, we have obtained a Ca^II^complex based on 2-ethyl-1*H*-imidazole-4,5-dicarboxylate under hydrothermal conditions (Tian *et al.*, 2010). In this paper, we report a new Zn^II^ complex.

The title compound, [Zn(C_7_H_7_N_2_O_4_)_2_ (H_2_O)_2_].2C_3_H_7_NO, as shown in Fig. 1, is a discrete complex, consisting of one Zn^II^ ion, two mono-deprotonated 2-ethyl-1*H*-imidazole-4,5-dicarboxy anions and two water molecules. Each Zn^II^ ion is six-coordinated in an octahedral coordination environment, formed by two oxygen atoms(O4,O4^i^) and two nitrogen atoms (N1,N1^i^)from two 2-ethyl-4,5-imidazoledicarboxylate ligands in the equatorial plane and two water molecules in the apical sites (symmetry codes: -*x* + 2, -*y*, -*z* + 1).the Zn—O bond distances are 2.1461 (18) Å and 2.2013 (18) Å, and Zn—N bond distances are 2.0683 (19) Å. Each 2-ethyl-4,5-imidazoledicarboxylate ligand chelates the Zn^II^ ion in a bidentate coordination mode through its imidazole nitrogen atom and carboxylate oxygen atom. Extensive hydrogen-bonding interactions (N—H···O and O—H···O), generate a two-dimensional structure.

### Experimental

A mixture of ZnNO_3_ (0.5 mmol, 0.06 g) and 2-ethyl-1*H*-imidazole-4,5-dicarboxylic acid(0.5 mmol, 0.95 g) dissolved in 10 ml C_3_H_7_NO, and then the solution was sealed in an autoclave equipped with a Teflon liner (25 ml) and then heated at 373k for 3 days. After slowly cooling down to room temperature, colourless crystals of the title compound were obtained directly from the solution.

### Refinement

Carbon and nitrogen bound H atoms were placed at calculated positions and were treated as riding on the parent C or N atoms with C—H = 0.93 Å, N—H = 0.86 Å, and with *U*_iso_(H) = 1.2 *U*_eq_(C, N). H atoms of the water molecule were located in a difference Fourier map and refined as riding with an O—H distance restraint of 0.84 (1) Å, with *U*_iso_(H) = 1.5 *U*_eq_. The H···H distances within the water molecules were restraint to 1.39 (1) Å. Carboxyl H atoms were located in a difference Fourier map and refined as riding with an O—H distance constraint of 0.85 Å, with *U*_iso_(H) = 1.2 *U*_eq_.

### Crystal data

**Table:**

[Zn(C_7_H_7_N_2_O_4_)_2_(H_2_O)_2_]·2C_3_H_7_NO	*F*(000) = 640
*M**_r_* = 613.89	*D*_x_ = 1.560 Mg m^−^^3^
Monoclinic, *P*2_1_/*c*	Mo *K*α radiation, λ = 0.71073 Å
Hall symbol: -P 2ybc	Cell parameters from 5837 reflections
*a* = 7.2817 (8) Å	θ = 2.8–27.9°
*b* = 20.660 (2) Å	µ = 1.01 mm^−^^1^
*c* = 9.3623 (9) Å	*T* = 296 K
β = 111.846 (7)°	Block, colourless
*V* = 1307.3 (2) Å^3^	0.53 × 0.41 × 0.31 mm
*Z* = 2	

### Data collection

**Table:**

Bruker APEXII area-detector diffractometer	2619 independent reflections
Radiation source: fine-focus sealed tube	2083 reflections with *I* > 2σ(*I*)
graphite	*R*_int_ = 0.037
φ and ω scan	θ_max_ = 26.2°, θ_min_ = 2.5°
Absorption correction: multi-scan (*SADABS*; Sheldrick, 1996)	*h* = −9→8
*T*_min_ = 0.616, *T*_max_ = 0.744	*k* = −25→25
10742 measured reflections	*l* = −11→11

### Refinement

**Table:**

Refinement on *F*^2^	Primary atom site location: structure-invariant direct methods
Least-squares matrix: full	Secondary atom site location: difference Fourier map
*R*[*F*^2^ > 2σ(*F*^2^)] = 0.034	Hydrogen site location: inferred from neighbouring sites
*wR*(*F*^2^) = 0.086	H-atom parameters constrained
*S* = 1.02	*w* = 1/[σ^2^(*F*_o_^2^) + (0.0387*P*)^2^ + 0.6624*P*] where *P* = (*F*_o_^2^ + 2*F*_c_^2^)/3
2619 reflections	(Δ/σ)_max_ < 0.001
181 parameters	Δρ_max_ = 0.22 e Å^−^^3^
3 restraints	Δρ_min_ = −0.36 e Å^−^^3^

### Special details

**Table:**

Geometry. All e.s.d.'s (except the e.s.d. in the dihedral angle between two l.s. planes) are estimated using the full covariance matrix. The cell e.s.d.'s are taken into account individually in the estimation of e.s.d.'s in distances, angles and torsion angles; correlations between e.s.d.'s in cell parameters are only used when they are defined by crystal symmetry. An approximate (isotropic) treatment of cell e.s.d.'s is used for estimating e.s.d.'s involving l.s. planes.
Refinement. Refinement of *F*^2^ against ALL reflections. The weighted *R*-factor *wR* and goodness of fit *S* are based on *F*^2^, conventional *R*-factors *R* are based on *F*, with *F* set to zero for negative *F*^2^. The threshold expression of *F*^2^ > σ(*F*^2^) is used only for calculating *R*-factors(gt) *etc*. and is not relevant to the choice of reflections for refinement. *R*-factors based on *F*^2^ are statistically about twice as large as those based on *F*, and *R*- factors based on ALL data will be even larger.

### Fractional atomic coordinates and isotropic or equivalent isotropic displacement parameters (Å^2^)

**Table:**

	*x*	*y*	*z*	*U*_iso_*/*U*_eq_	
Zn1	1.0000	0.0000	1.0000	0.02973 (13)	
O1	0.6355 (3)	−0.00930 (9)	0.25342 (18)	0.0414 (4)	
O1W	1.2808 (2)	0.03585 (9)	1.00911 (17)	0.0383 (4)	
H1W	1.2982	0.0255	0.9272	0.057*	
H2W	1.3729	0.0197	1.0863	0.057*	
O2	0.7948 (3)	−0.09580 (9)	0.38356 (18)	0.0424 (4)	
O3	0.9815 (3)	−0.12960 (8)	0.65214 (18)	0.0392 (4)	
H3	0.9214	−0.1176	0.5595	0.047*	
O4	1.0464 (2)	−0.08767 (8)	0.88405 (17)	0.0358 (4)	
N1	0.8675 (3)	0.02603 (9)	0.77101 (19)	0.0263 (4)	
N2	0.7049 (3)	0.05738 (9)	0.5331 (2)	0.0287 (4)	
H2	0.6373	0.0814	0.4565	0.034*	
C1	0.7687 (3)	0.07446 (11)	0.6824 (2)	0.0280 (5)	
C2	0.8660 (3)	−0.02367 (11)	0.6730 (2)	0.0247 (4)	
C3	0.7660 (3)	−0.00476 (11)	0.5237 (2)	0.0274 (5)	
C4	0.9705 (3)	−0.08386 (11)	0.7418 (2)	0.0292 (5)	
C5	0.7267 (3)	−0.03848 (12)	0.3751 (2)	0.0326 (5)	
C6	0.7387 (4)	0.14021 (12)	0.7351 (3)	0.0407 (6)	
H6A	0.7823	0.1403	0.8464	0.049*	
H6B	0.5986	0.1504	0.6934	0.049*	
C7	0.8514 (5)	0.19225 (14)	0.6854 (4)	0.0594 (8)	
H7A	0.9895	0.1815	0.7230	0.089*	
H7B	0.8342	0.2333	0.7269	0.089*	
H7C	0.8013	0.1947	0.5751	0.089*	
O5	0.4712 (3)	0.14244 (10)	0.32830 (19)	0.0504 (5)	
N3	0.4143 (3)	0.18961 (10)	0.0978 (2)	0.0412 (5)	
C8	0.5131 (4)	0.15203 (13)	0.2144 (3)	0.0428 (6)	
H8A	0.6239	0.1309	0.2104	0.051*	
C9	0.4683 (6)	0.1927 (2)	−0.0361 (4)	0.0754 (10)	
H9A	0.5986	0.1751	−0.0109	0.113*	
H9B	0.4665	0.2369	−0.0679	0.113*	
H9C	0.3755	0.1680	−0.1182	0.113*	
C10	0.2338 (5)	0.22132 (16)	0.0909 (4)	0.0654 (9)	
H10A	0.2220	0.2184	0.1895	0.098*	
H10B	0.1223	0.2005	0.0147	0.098*	
H10C	0.2374	0.2660	0.0640	0.098*	

### Atomic displacement parameters (Å^2^)

**Table:**

	*U*^11^	*U*^22^	*U*^33^	*U*^12^	*U*^13^	*U*^23^
Zn1	0.0328 (2)	0.0381 (2)	0.01527 (18)	0.00315 (17)	0.00554 (15)	0.00143 (15)
O1	0.0407 (10)	0.0605 (12)	0.0179 (8)	0.0054 (8)	0.0051 (8)	0.0017 (8)
O1W	0.0322 (9)	0.0579 (12)	0.0227 (8)	−0.0008 (8)	0.0078 (7)	0.0013 (8)
O2	0.0510 (11)	0.0429 (11)	0.0271 (9)	0.0046 (9)	0.0075 (8)	−0.0077 (7)
O3	0.0489 (10)	0.0363 (9)	0.0271 (9)	0.0113 (8)	0.0079 (8)	−0.0007 (7)
O4	0.0433 (10)	0.0374 (9)	0.0211 (8)	0.0090 (8)	0.0055 (7)	0.0040 (7)
N1	0.0285 (10)	0.0322 (10)	0.0167 (9)	0.0036 (8)	0.0065 (8)	0.0015 (7)
N2	0.0295 (10)	0.0336 (11)	0.0196 (9)	0.0060 (8)	0.0053 (8)	0.0066 (8)
C1	0.0283 (12)	0.0331 (12)	0.0218 (11)	0.0025 (9)	0.0082 (10)	0.0026 (9)
C2	0.0250 (11)	0.0297 (11)	0.0183 (10)	0.0009 (9)	0.0068 (9)	0.0007 (8)
C3	0.0251 (11)	0.0359 (12)	0.0204 (10)	−0.0016 (10)	0.0075 (9)	0.0005 (9)
C4	0.0292 (12)	0.0323 (12)	0.0252 (12)	0.0008 (10)	0.0092 (10)	−0.0001 (9)
C5	0.0278 (12)	0.0450 (15)	0.0230 (12)	−0.0046 (11)	0.0072 (10)	−0.0024 (10)
C6	0.0472 (15)	0.0407 (15)	0.0313 (13)	0.0133 (12)	0.0114 (12)	−0.0014 (11)
C7	0.067 (2)	0.0383 (16)	0.064 (2)	−0.0033 (15)	0.0142 (17)	−0.0061 (14)
O5	0.0557 (12)	0.0593 (13)	0.0310 (10)	0.0156 (10)	0.0101 (9)	0.0128 (8)
N3	0.0462 (12)	0.0405 (12)	0.0304 (11)	0.0003 (10)	0.0069 (10)	0.0045 (9)
C8	0.0419 (15)	0.0422 (15)	0.0390 (15)	0.0083 (12)	0.0090 (12)	0.0028 (12)
C9	0.091 (3)	0.095 (3)	0.0440 (18)	−0.008 (2)	0.0296 (19)	0.0128 (17)
C10	0.066 (2)	0.062 (2)	0.0517 (18)	0.0231 (17)	0.0028 (16)	0.0102 (15)

### Geometric parameters (Å, °)

**Table:**

Zn1—N1	2.0680 (17)	C2—C4	1.475 (3)
Zn1—N1^i^	2.0680 (17)	C3—C5	1.485 (3)
Zn1—O1W^i^	2.1464 (16)	C6—C7	1.526 (4)
Zn1—O1W	2.1464 (16)	C6—H6A	0.9700
Zn1—O4	2.2013 (16)	C6—H6B	0.9700
Zn1—O4^i^	2.2013 (16)	C7—H7A	0.9600
O1—C5	1.241 (3)	C7—H7B	0.9600
O1W—H1W	0.8501	C7—H7C	0.9600
O1W—H2W	0.8500	O5—C8	1.230 (3)
O2—C5	1.275 (3)	N3—C8	1.314 (3)
O3—C4	1.286 (3)	N3—C10	1.448 (4)
O3—H3	0.8500	N3—C9	1.448 (3)
O4—C4	1.240 (3)	C8—H8A	0.9300
N1—C1	1.328 (3)	C9—H9A	0.9600
N1—C2	1.375 (3)	C9—H9B	0.9600
N2—C1	1.346 (3)	C9—H9C	0.9600
N2—C3	1.372 (3)	C10—H10A	0.9600
N2—H2	0.8600	C10—H10B	0.9600
C1—C6	1.489 (3)	C10—H10C	0.9600
C2—C3	1.371 (3)		
			
N1—Zn1—N1^i^	180.0	O4—C4—C2	118.3 (2)
N1—Zn1—O1W^i^	88.77 (7)	O3—C4—C2	118.78 (19)
N1^i^—Zn1—O1W^i^	91.23 (6)	O1—C5—O2	124.8 (2)
N1—Zn1—O1W	91.23 (6)	O1—C5—C3	118.9 (2)
N1^i^—Zn1—O1W	88.77 (7)	O2—C5—C3	116.3 (2)
O1W^i^—Zn1—O1W	180.0	C1—C6—C7	112.3 (2)
N1—Zn1—O4	78.50 (6)	C1—C6—H6A	109.1
N1^i^—Zn1—O4	101.50 (6)	C7—C6—H6A	109.1
O1W^i^—Zn1—O4	90.87 (6)	C1—C6—H6B	109.1
O1W—Zn1—O4	89.13 (6)	C7—C6—H6B	109.1
N1—Zn1—O4^i^	101.50 (6)	H6A—C6—H6B	107.9
N1^i^—Zn1—O4^i^	78.50 (6)	C6—C7—H7A	109.5
O1W^i^—Zn1—O4^i^	89.13 (6)	C6—C7—H7B	109.5
O1W—Zn1—O4^i^	90.87 (6)	H7A—C7—H7B	109.5
O4—Zn1—O4^i^	180.000 (1)	C6—C7—H7C	109.5
Zn1—O1W—H1W	109.7	H7A—C7—H7C	109.5
Zn1—O1W—H2W	109.7	H7B—C7—H7C	109.5
H1W—O1W—H2W	109.5	C8—N3—C10	120.8 (2)
C4—O3—H3	108.6	C8—N3—C9	120.2 (3)
C4—O4—Zn1	112.88 (14)	C10—N3—C9	118.4 (2)
C1—N1—C2	106.13 (17)	O5—C8—N3	125.4 (2)
C1—N1—Zn1	141.17 (15)	O5—C8—H8A	117.3
C2—N1—Zn1	112.51 (14)	N3—C8—H8A	117.3
C1—N2—C3	108.49 (18)	N3—C9—H9A	109.5
C1—N2—H2	125.8	N3—C9—H9B	109.5
C3—N2—H2	125.8	H9A—C9—H9B	109.5
N1—C1—N2	110.41 (19)	N3—C9—H9C	109.5
N1—C1—C6	126.4 (2)	H9A—C9—H9C	109.5
N2—C1—C6	123.2 (2)	H9B—C9—H9C	109.5
C3—C2—N1	109.71 (19)	N3—C10—H10A	109.5
C3—C2—C4	132.7 (2)	N3—C10—H10B	109.5
N1—C2—C4	117.60 (18)	H10A—C10—H10B	109.5
C2—C3—N2	105.25 (18)	N3—C10—H10C	109.5
C2—C3—C5	131.7 (2)	H10A—C10—H10C	109.5
N2—C3—C5	123.0 (2)	H10B—C10—H10C	109.5
O4—C4—O3	122.9 (2)		

Symmetry codes: (i) −*x*+2, −*y*, −*z*+2.

### Hydrogen-bond geometry (Å, °)

**Table:**

*D*—H···*A*	*D*—H	H···*A*	*D*···*A*	*D*—H···*A*
O1W—H1W···O1^ii^	0.85	1.95	2.798 (2)	173
O1W—H2W···O1^iii^	0.85	2.06	2.894 (2)	168
O3—H3···O2	0.85	1.62	2.473 (2)	177
N2—H2···O5	0.86	1.85	2.689 (2)	166

Symmetry codes: (ii) −*x*+2, −*y*, −*z*+1; (iii) *x*+1, *y*, *z*+1.
